# A Cytosolic Phosphoglucose Isomerase, OsPGI1c, Enhances Plant Growth and Herbivore Resistance in Rice

**DOI:** 10.3390/ijms26010169

**Published:** 2024-12-28

**Authors:** Lin Chen, Peng Kuai, Jing Lu, Leilei Li, Yonggen Lou

**Affiliations:** 1 College of Plant Protection, Yangzhou University, Yangzhou 225009, China; chenlin88@yzu.edu.cn; 2 State Key Laboratory of Rice Biology & Ministry of Agriculture Key Laboratory of Molecular Biology of Crop Pathogens and Insects, Institute of Insect Sciences, Zhejiang University, Hangzhou 310058, China; kpchen7493@163.com (P.K.); jing_lu@zju.edu.cn (J.L.); lileilei@zju.edu.cn (L.L.)

**Keywords:** *Oryza sativa*, cytosolic phosphoglucose isomerase, *OsPGI1c*, growth, brown planthopper, herbivore-induced defenses

## Abstract

Glucose-6-phosphate isomerase (PGI), a key enzyme that catalyzes the reversible conversion of glucose-6-phosphate and fructose-6-phosphate, plays an important role in plant growth, development, and responses to abiotic stresses and pathogen infections. However, whether and how PGI modulates herbivore-induced plant defenses remain largely unknown. The Brown planthopper (BPH, *Nilaparvata lugens*) is a devastating insect pest of rice, causing significant damage to rice plants through feeding, oviposition, and disease transmission, resulting in great yield losses. Here, we isolated a rice cytosolic PGI gene, *OsPGI1c*, which is ubiquitously expressed in rice plants; the highest transcript levels are found in leaves, outer leaf sheaths, and seeds. The expression of *OsPGI1c* was induced by infestation by gravid females of the BPH, mechanical wounding, and treatment with jasmonic acid (JA). Overexpressing *OsPGI1c* in rice (oePGI) enhanced both the masses of plant shoots and roots and basal levels of trehalose; however, when infested by gravid BPH females for 2 days, trehalose levels were significantly lower in oePGI plants than in wild-type (WT) plants. Additionally, the overexpression of *OsPGI1c* increased the BPH-induced levels of JA, jasmonoyl-L-isoleucine, and abscisic acid, but decreased the levels of ethylene and H_2_O_2_. Bioassays revealed that gravid BPH females preferred WT plants over oePGI plants for laying eggs; moreover, BPH eggs exhibited lower hatching rates and required longer developmental durations on oePGI plants than WT plants. These results indicate that OsPGI1c positively modulates both rice growth and BPH resistance.

## 1. Introduction

Upon attack by insect herbivores, plants recognize herbivore-associated molecular patterns through specific receptors and rapidly activate early signaling events, such as mitogen-activated protein kinase (MAPK) cascades and reactive oxygen species bursts, as well as signaling pathways mediated by phytohormones, such as jasmonic acid (JA), salicylic acid (SA), abscisic acid (ABA), and ethylene (ET) [1,2,3,4]. These activated phytohormone-signaling pathways lead to changes in the plant transcriptomes, which in turn alter the metabolome and enhance the resistance of plants to herbivores [5]. On the other hand, primary metabolites play an important role in plant responses to insect herbivores, as they provide energy for defense and serve as precursors for many defense-related metabolites [6,7]. Moreover, primary metabolites can function as signaling molecules in herbivore-induced plant defense processes [6,7]. For example, herbivory by *Helicoverpa zea and Manduca sexta* strongly induces changes in primary metabolites in tomato (*Solanum lycopersicum*), which are associated with plant resistance and tolerance [8]. Machado et al. [9] reported that jasmonates suppressed the activity of soluble invertases and reduced herbivore-induced glucose and fructose concentrations in the leaves of *Nicotiana attenuate*. This suppression of sugars led to the decreased resistance of *N. attenuate* to *M. sexta* [9].

Phosphoglucose isomerase (PGI) (EC 5.3.1.9), also known as glucose-6-phosphate isomerase, is a dimeric enzyme that catalyzes the reversible conversion of glucose-6-phosphate (G6P) and fructose-6-phasphate (F6P) [10]. This enzymatic reaction plays a critical role in carbohydrate metabolism, such as glycolysis [11], starch biosynthesis [10], and the oxidative pentose phosphate pathway (OPPP) [12]. In general, there are two types of PGI isoforms in higher plants: the cytosolic PGI (cPGI) and the plastidic PGI (pPGI) [13]. These two isoforms are encoded by different nuclear genes, *PGIc* and *PGIp* [14]. In the *Arabidopsis* genus, there is only a single *PGI* gene for each isoform [15], whereas in rice, there are two *PGIc* genes and one *PGIp* gene [16]. PGI is an important determinant of plant growth, development, and reproduction [17,18]. For instance, a T-DNA insertion pPGI mutant *pgi1-2* in *Arabidopsis thaliana* accumulates low amounts of starch and cytokinins derived from plastidic 2-C-methyl-D-erythritol 4-phosphate-pathway; moreover, the mutant shows a retarded growth phenotype and reduced photosynthetic capacity [19]. The overexpression of a wheat *cPGI* in the chloroplasts of an Arabidopsis *atpgip* mutant enhanced starch accumulation, CO_2_ assimilation, plant biomass, and seed yield productivity [20]. Additionally, Kunz et al. [15] reported that Arabidopsis heterozygous *cPGI* T-DNA mutants produce more empty spots in siliques and exhibit significantly decreased transmission efficiency through male and female gametophytes; moreover, Arabidopsis *cpgi* knockout mutants display impaired young vegetative growth as well as reduced female fertility and male sterility [21].

In addition to its vital role in plant physiological processes, PGI is reportedly involved in plants’ responses to both abiotic and biotic stresses. Cui et al. [22,23] reported that PGI is a salt-induced protein in the green algae *Dunaliella salina*: the higher the salinity of the surrounding water, the higher the *PGI* expression level of the algae. In maize, anaerobic stress strongly enhances the enzymatic activity of protein ANP55, a cytosolic PGI [24,25]. The transient overexpression of Chinese silvergrass (*Miscanthus sinensis*) *PGI* (*MsPGI*) in *Nicotiana benthamiana* led to the increased expression of ascorbate peroxidase and phenylalanine ammonia lyase, suggesting that *MsPGI* may be involved in antioxidant metabolism [26]. Moreover, a proteomic analysis of fungal elicitor-treated *Arabidopsis* cell cultures has also identified cytosolic PGI as a protein responsive to fungal elicitors [27]. In summary, although PGI plays a role in plants against abiotic stresses and may be involved in pathogen-induced plant defenses, whether PGI regulates defense responses in plants against herbivorous insects remains largely unknown.

Rice, the world’s most important staple food, feeds more than half its population, especially in Asia [28]. The brown planthopper (BPH, *Nilaparvata lugens*) is one of the most important insect pests on rice; it damages rice plants by feeding on phloem sap, ovipositing in plant tissues, and transmitting viruses [29,30]. Previous research has shown that BPH infestation can activate the signaling pathways mediated by JA, SA, ET, ABA, and H_2_O_2_ in rice; these pathways, in turn, cause changes in the plant’s metabolomes and thus, regulate its resistance to BPH [30,31,32,33]. JA-, H_2_O_2_- and ABA-mediated pathways have been reported to positively modulate the resistance of rice to piercing and sucking insects such as BPH [34,35,36], whereas the ET-mediated pathways negatively regulate the resistance of rice to BPH [37]. Besides secondary compounds, such as phenolamides and phenolic compounds [38,39], some primary compounds, such as amino acids and sugars, have also been reported to play important roles in modulating BPH performance [40,41,42,43].

Given the critical role of PGI in carbohydrate metabolism and the importance of carbohydrates in plant defenses, we cloned a cytosolic PGI, designated *OsPGI1c*, which was found to be induced by herbivore infestation [44], and characterized its role in herbivore resistance in rice. Our results suggest that *OsPGI1c* positively regulates rice defense against BPH by up-regulating BPH-induced accumulation levels of phytohormones JA, ET, and ABA. Moreover, *OsPGI1c* also positively modulates rice growth.

## 2. Results

### 2.1. Isolation and Sequence Analysis of OsPGI1c

**Figure 1:**
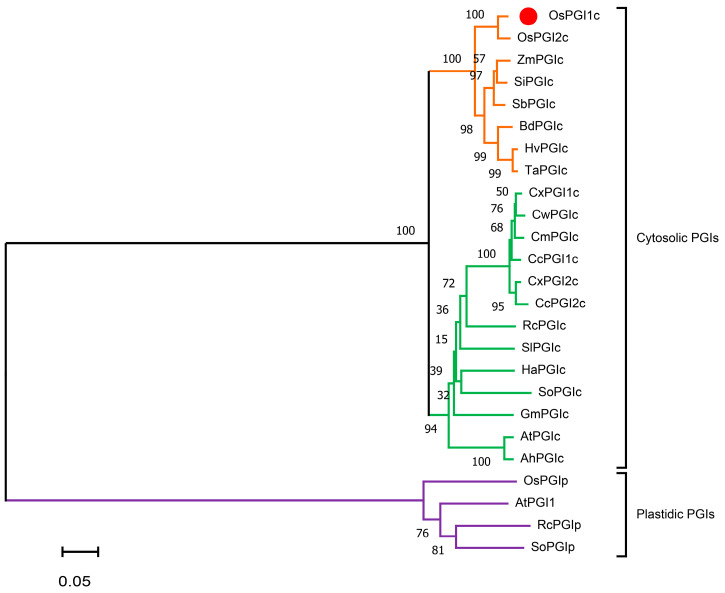
Phylogenic analysis of the OsPGI1c protein and its homologs from other higher plant species. The amino acid sequences of PGIs were aligned using ClustalW and subjected to phylogenetic analysis with the Mega 11 software. OsPGI1c is marked with a red circle in the phylogenetic tree. Detailed information about the plant PGI proteins is provided in Table S1.

We cloned the full-length coding sequence (CDS) from a rice stem cDNA library (variety XS110) of the G6P isomerase gene (TIGR ID: Os03g56460) (Figure S1). The full-length CDS of *OsPGI1c* is 1704 bp, encoding a putative protein of 567 amino acids with a theoretical molecular weight of 62.5 kD and a predicted isoelectric point of 7.3 (Figure S2). A sequence analysis of the amino acids demonstrated that OsPGI1c contains two sugar isomerase (SIS) domains (Figure S1). To further analyze the evolutionary relationship among OsPGI1c and other PGIs, a phylogenic tree of 25 plant PGI proteins was constructed. The plant PGIs are clearly clustered into two clades: cytosolic and plastidic PGIs (Figure 1). According to the phylogenetic tree, OsPGI1c belongs to the cytosolic PGI group. Within this group, OsPGI1c and PGIs from the Poaceae family plants, including OsPGI2c, ZmPGIc in Maize (*Zea mays*), SiPGIc in foxtail millet (*Setaria italica*), SbPGIc in sorghum (*Sorghum bicolor*), BdPGIc in false brome (*Brachypodium distachyon*), HvPGIc in barley (*Hordeum vulgare*), and TaPGIc in wheat (*Triticum aestivum*), are highly similar, with an identity of 97.18%, 91.18%, 91.18%, 90.48%, 91.01%, 89.24%, and 89.21%, respectively (Figure 1, Figure S3). Multiple sequence alignments of the Poaceae PGI proteins further confirmed that the amino acid sequences of PGIs are highly conserved across these plants (Figure S3). These results demonstrated that OsPGI1c is a cytosolic isomerase.

### 2.2. Tissue Expression Patterns of OsPGI1c

**Figure 2:**
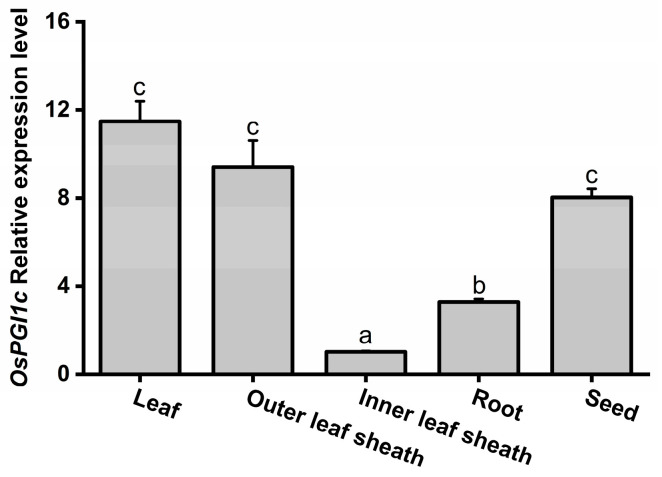
Relative expression levels of *OsPGI1c* in rice seeds, as well as in leaves, outer leaf sheaths, inner leaf sheaths, and roots of 30-day-old WT plants. Data are means + standard error (*n* = 6). Different letters indicate significant differences in relative expression levels among the various tissues determined by a one-way ANOVA followed by Tukey’s HSD post hoc test (*p* < 0.05).

To examine the expression of *OsPGI1c* in various rice tissues, total RNA was isolated from 30-day-old rice plants and seeds, and quantitative real-time PCR (qRT-PCR) was performed. The results showed that *OsPGI1c* was expressed in all five tissues at varying transcript levels. The highest levels of *OsPGI1c* transcripts were observed in leaves, outer leaf sheaths, and seeds, followed by roots; the lowest levels were detected in inner leaf sheaths (Figure 2).

### 2.3. BPH Infestation and JA Treatment Induce the Expression of OsPGI1c

**Figure 3:**
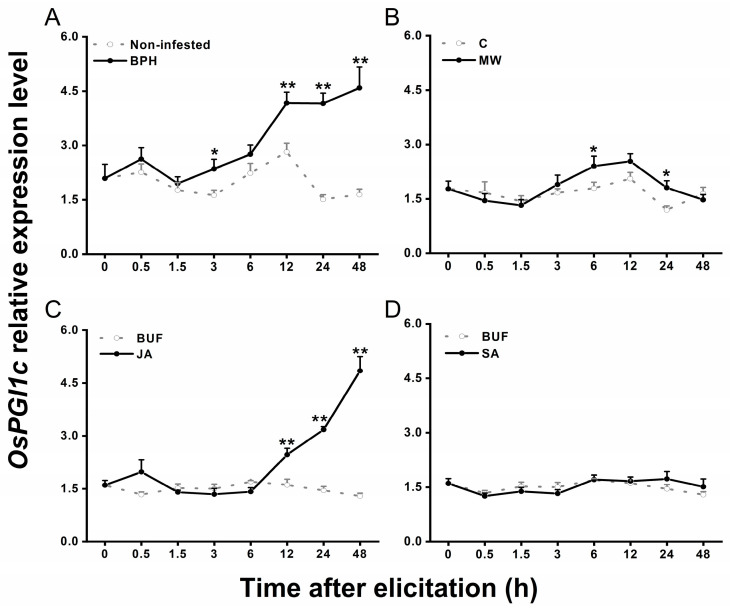
Relative expression levels of *OsPGI1c* in the leaf sheaths of 30-day-old wild-type plants subjected to different treatments. (**A**), infestation by 15 gravid BPH females; (**B**), mechanical wounding; (**C**), treatment with JA; (**D**), treatment with SA. Data are means + standard error (*n* = 6). Asterisks indicate significant differences between treatments and controls (*, *p* < 0.05; **, *p* < 0.01; Student’s *t*-test).

To check whether *OsPGI1c* is involved in BPH-induced rice defenses, we investigated the transcript level of *OsPGI1c* in plants before and after they were infested by gravid BPH females. Compared to the transcript level of *OsPGI1c* in the non-infested rice, BPH infestation significantly increased the level in plants starting at 3 h and peaking at 48 h post-BPH infestation (Figure 3A). Mechanical wounding only slightly induced the expression of *OsPGI1c* at 6 and 24 h post-elicitation (Figure 3B). The JA treatment significantly enhanced the transcript level of *OsPGI1c* 12-48 h after treatment, whereas the SA treatment had no effect (Figure 3C,D). These results suggest that *OsPGI1c* may be involved in rice defense responses to BPH.

### 2.4. Overexpression of OsPGI1c Promotes Rice Growth

**Figure 4:**
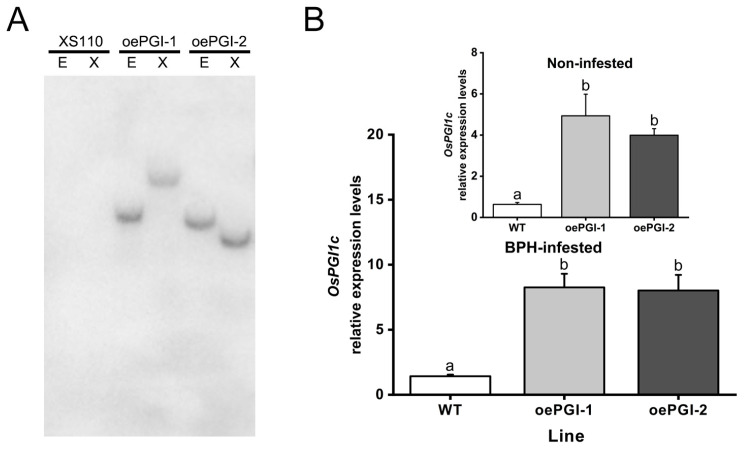
Molecular confirmation of lines overexpressing *OsPGI1c*. (**A**) Southern blot analysis of transgenic plants. The genomic DNA of oePGI and WT plants was extracted and digested with restriction enzyme *EcoR* I (E) or *Xbal* I (X), and then used for hybridization. (**B**) The relative expression levels of *OsPGI1c* in the leaf sheaths of oePGI (oePGI-1 and oePGI-2) and WT plants 12 h after infestation by 15 gravid BPH females. Insert: levels of *OsPGI1c* transcripts in the oePGI and WT plants not infested by BPH. Data are means + standard error (*n* = 5). Different letters indicate significant differences in oePGI lines compared with the WT plants determined by a one-way ANOVA followed by Tukey’s HSD post hoc test (*p* < 0.05).

To explore the role of *OsPGI1c* in interactions between rice and BPH, we generated rice lines overexpressing *OsPGI1c* (oePGI lines) using an *Agrobacterium*-based transformation system. Two T_2_ transgenic homozygous lines, designated oePGI-1 and oePGI-2, each with a single insertion (Figure 4A), were obtained. The qRT-PCR analysis revealed that both the basal and BPH-induced (12 h after BPH infestation) transcript levels of *OsPGI1c* were significantly higher in the two oePGI lines than in the wild-type (WT) plants (Figure 4B and insert).

**Figure 5:**
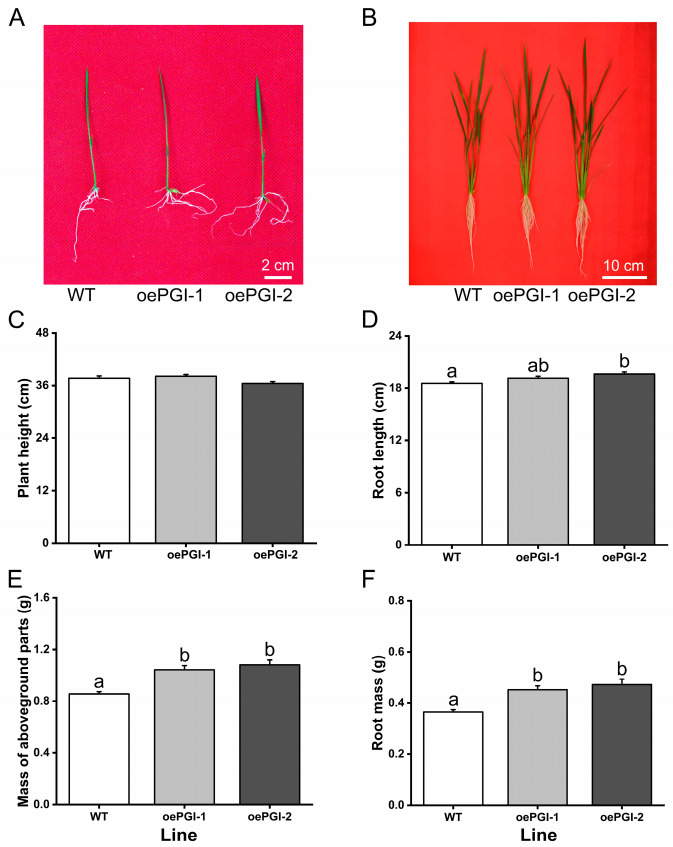
Growth phenotype of WT and oePGI plants. (**A**,**B**) Photos showing 10-day-old (**A**) and 30-day-old (**B**) plants of WT and oePGI lines. (**C**–**F**) Plant height, root length, and mass of both aboveground parts and roots of 30-day-old plants of WT and oePGI lines. Data are means + standard error (*n* = 20). Different letters indicate significant differences in oePGI lines compared with WT plants determined by one-way ANOVA followed by Tukey’s HSD post hoc test (*p* < 0.05).

No obvious difference in growth phenotypes was observed between the WT and oePGI plants at the 10-day stage (Figure 5A), nor in plant height at the 30-day stage (Figure 5B,C). However, while only a slight difference in root length was found between the WT and transgenic plants (Figure 5D), higher shoot and root masses were found in the oePGI plants than in the WT plants at the 30-day stage (Figure 5E,F). These data suggest that *OsPGI1c* promotes rice growth.

### 2.5. Overexpressing OsPGI1c Alters the Accumulation of Trehalose but Not Sucrose, Glucose, and Fructose

**Figure 6:**
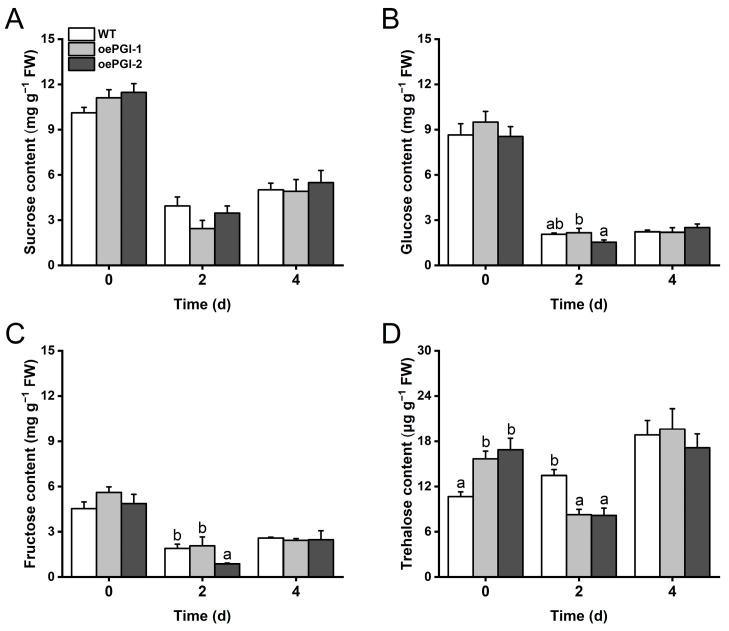
Levels of sucrose (**A**), glucose (**B**), fructose (**C**), and trehalose (**D**) in oePGI and WT plants at different time points after gravid BPH female infestation. Data are means + standard error (*n* = 6). Different letters represent significant differences between WT and transgenic plants determined by one-way ANOVA followed by Tukey’s HSD post hoc test (*p* < 0.05).

Given the importance of cPGI in mediating carbohydrate metabolism, as mentioned above, we measured the contents of soluble sugars, including sucrose, glucose, fructose, and trehalose in the oePGI and WT plants before and after they were infested by gravid BPH females. The levels did not differ significantly between the oePGI and WT plants at 0, 2, and 4 days after BPH infestation, although a significant decrease in the levels of fructose was observed in oePGI-2 at 2 days after BPH infestation (Figure 6A–C). Intriguingly, we found that whereas the basal level of trehalose was higher in the oePGI plants compared to the WT plants, 2 days after BPH infestation, it was significantly lower (Figure 6D).

### 2.6. OsPGI1c Regulates BPH-Induced Accumulation of JA, JA-Ile, ABA, and Ethylene

**Figure 7:**
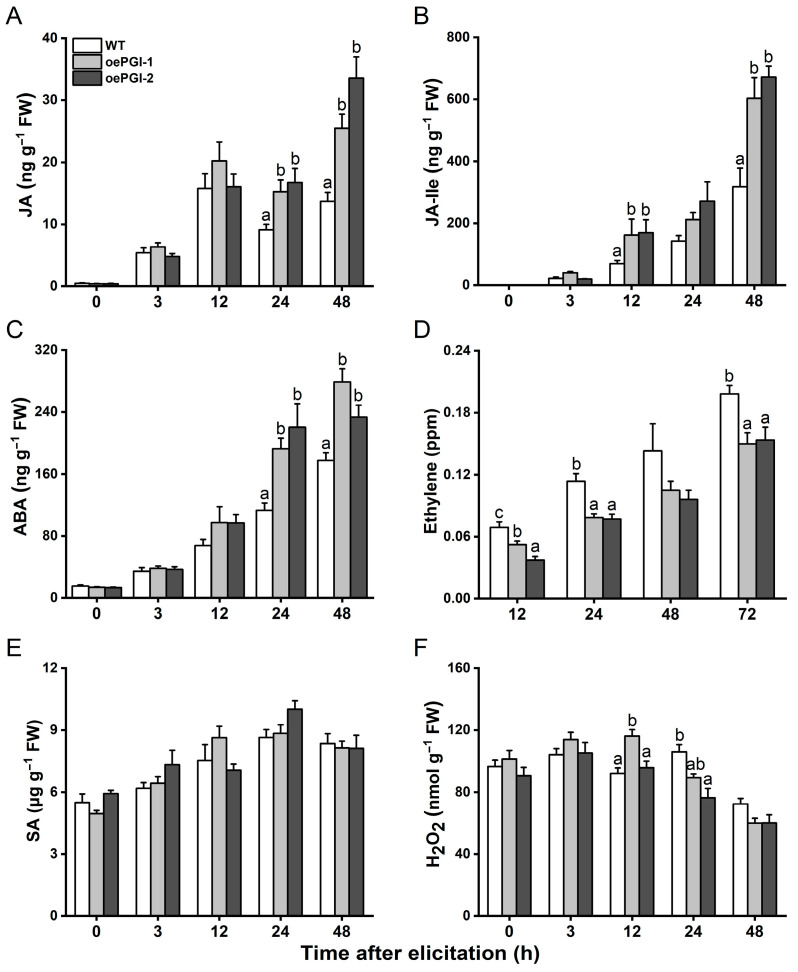
Levels of JA (**A**), JA-Ile (**B**), ABA (**C**), ethylene (**D**), SA (**E**), and H_2_O_2_ (**F**) in WT and oePGI lines at different time points after they were infested by 15 gravid BPH females. Data are means + standard error (*n* = 6). Different letters represent significant differences between WT and transgenic plants determined by one-way ANOVA followed by Tukey’s HSD post hoc test (*p* < 0.05).

Signaling pathways mediated by JA, JA-Ile, ABA, ethylene, SA, and H_2_O_2_ play a central role in herbivore-induced rice defenses [34,35,36,37]. Thus, we asked if overexpressing *OsPGI1c* influences the BPH-induced levels of these signaling molecules in plants. No significant differences were observed in the basal levels of JA and JA-Ile between the WT and oePGI plants (Figure 7A,B). However, when the plants were infested by gravid BPH females, both JA (at 24 and 48 h after infestation) and JA-Ile (at 12 and 48 h after infestation) levels were significantly higher in the oePGI lines than in the WT plants (Figure 7A,B). Similarly, although there was no difference in the basal ABA levels, the BPH-elicited levels of ABA were significantly higher in the oePGI lines than in the WT plants at 24 and 48 h post-infestation (Figure 7C). In contrast, the oePGI plants released less ethylene than did the WT plants at 12, 24, 48, and 72 h post-BPH infestation, although the difference is not significant at 48 h (Figure 7D). Overexpressing *OsPGI1c* had no impact on the basal and BPH-induced SA levels at any time points (Figure 7E) and only a slight impact (decrease) on the BPH-induced H_2_O_2_ levels at 24 and 48 h after infestation, although one oePGI line exhibited higher levels compared to the WT plants at 12 h (Figure 7F).

### 2.7. Overexpression of OsPGI1c Enhances the Resistance of Rice to BPH

**Figure 8:**
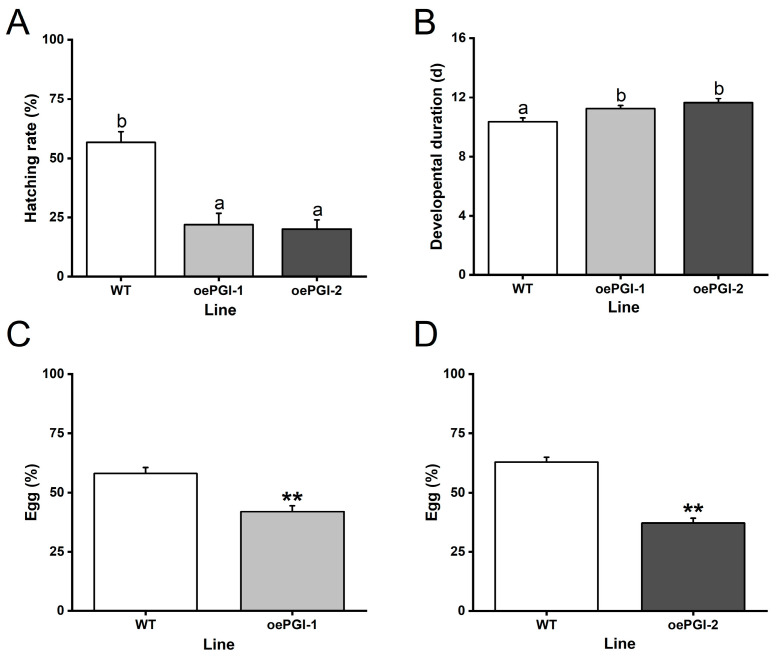
Overexpression of *OsPGI1c* enhanced the resistance of rice to BPH. Data are means + standard error (*n* = 10). Hatching rates (**A**) and developmental durations (**B**) of BPH eggs laid on the oePGI and WT plants. Different letters indicate significant differences in the oePGI lines compared with the WT plants determined by one-way ANOVA followed by Tukey’s HSD post hoc test (*p* < 0.05). (**C**,**D**) The percentages of BPH eggs laid on each plant of a pair (a WT plant and an oePGI-1 or oePGI-2 plant). Asterisks indicate significant differences in the oePGI lines compared with the WT plants determined by a *Chi*-square test (**, *p* <0.01).

Since overexpressing *OsPGI1c* enhanced the BPH-induced levels of JA, JA-Ile, and ABA, we further investigated the effect of overexpressing *OsPGI1c* in rice on the survival and development of BPH eggs. The results revealed that the overexpression of *OsPGI1c* significantly reduced the hatching rate of BPH eggs: the hatching rates of BPH eggs laid on the oePGI-1 and oePGI-2 plants were only 38.8% and 35.3%, respectively, of those of BPH eggs laid on the WT plants (Figure 8A). Additionally, overexpressing *OsPGI1c* also prolonged the developmental duration of BPH eggs: the developmental durations of BPH eggs laid on the two oePGI lines, oePGI-1 and oePGI-2, were 8.7% and 12.4% longer, respectively, than that on the WT plants (Figure 8B). Furthermore, we found that gravid BPH females preferred to lay eggs on the WT plants over the oePGI plants (Figure 8C,D). These findings suggest that overexpressing *OsPGI1c* can potentially enhance rice resistance to BPH.

## 3. Discussion

In this study, a rice cytosolic glucose-6-phosphate isomerase gene, *OsPGI1c,* was cloned and characterized. We found that *OsPGI1c* was expressed in all five examined tissues, with the highest transcript levels in leaves and leaf sheaths (Figure 2), and could be moderately induced by gravid BPH female infestation and JA treatment (Figure 3). Overexpressing *OsPGI1c* increased plant shoot and root masses (Figure 4 and Figure 5) as well as the basal levels of trehalose in rice, but reduced the BPH-induced levels of trehalose at 2 d post-BPH infestation (Figure 6). Moreover, the overexpression of *OsPGI1c* enhanced the BPH-induced levels of JA, JA-Ile, and ABA in rice plants while decreasing ethylene levels (Figure 7); these results, in turn, both decreased the hatching rates of BPH eggs and the oviposition preference of gravid BPH females for the plants, as well as prolonged the developmental duration of BPH eggs (Figure 8), suggesting that *OsPGI1c* positively regulates rice growth and resistance to BPH.

PGIc has been reported to be expressed mainly in leaves and developing embryos [21]. In sunflower (*Helianthus annuus* L.), for example, at its initial developmental stages, *HacPGI* exhibited the highest expression levels in seeds and leaves [45]. Similarly, Arabidopsis *PGIc* transcripts accumulated abundantly in leaves and developing embryos and seeds [15]. Here, we also observed that the levels of *OsPGI1c* transcripts are the highest in rice leaves, outer leaf sheaths, and seeds. PGIc catalyzes the reversible interconversion of G6P and F6P, which is a core reaction in the anabolism and catabolism of sucrose [18]. As carbohydrates are synthesized in leaves and reserved in the leaf sheaths and seeds in rice [46], the high expression of *OsPGI1c* in rice leaves, outer leaf sheaths, and seeds may reflect high glycolytic or gluconeogenic rates in these tissues, supporting the optimal growth, development, and reproduction [47,48]. *PGI* can reportedly be induced by abiotic and biotic stresses [22,25,27]. Chivasa et al. [27], for example, found that Arabidopsis *cPGI* was up-regulated when plants were treated with fungal elicitors. In *Miscanthus sinensis*, treatments with NaCl or ABA strongly induced the expression of *MsGPI*, whereas mannitol and methyl viologen, which mimic drought and oxidative stresses, respectively, suppressed its expression [26]. Similarly, in this study, we observed that BPH adult female infestation, mechanical wounding, and the JA treatment significantly induced the expression of *OsPGI1c,* whereas the SA treatment did not. Additionally, previous studies have demonstrated that BPH infestation activates the JA-signaling pathway, but not the SA-signaling pathway [36,49], suggesting that *OsPGI1c* is involved in the rice responses to BPH through the JA-signaling pathway.

That overexpressing *OsPGI1c* increased the mass of plant shoots and roots (Figure 5) aligns with previous reports in other plants. In Arabidopsis, for instance, lines overexpressing an artificial microRNA (*amiRNA*) targeting *cPGI* showed a significantly diminished growth phenotype, including reduced leaf area and low fresh masses of leaves [15]. Recently, Gao et al. [20] reported that the transformation of *TaPGIc* (from wheat) into the chloroplasts of the Arabidopsis *atpgip* mutant enhanced photosynthesis rates, biomass, and yield. Meanwhile, the antisense repression of the gene encoding the potato cytosolic phosphoglucomutase (StcPGM), an enzyme immediately downstream of the cPGI, severely stunted plant phenotypes and shortened root lengths [50]; *amiRNA* transgenic plants lacking the entire cPGM activity in Arabidopsis resulted in a strong growth reduction, which decreased the fresh weight of rosettes and shortened roots [51]. The change in the growth phenotypes of these mutants may be attributed to the central roles of cPGI and cPGM in the anabolism and catabolism of carbohydrates [52]. In Arabidopsis, for example, the loss of *cPGI* reduced the levels of leaf sucrose and increased the concentration of glucose by the end of the night, whereas the levels of leaf sucrose were unchanged during the light period [15]. However, in this study, we did not find significant differences between the levels of soluble sugars, such as sucrose, glucose, and fructose, in the rice leaf sheaths of the oePGI and WT plants during the light period (Figure 6A–C). This discrepancy may be related to sampling time and tissues. In addition, phosphorylated sugars such as G6P have been shown to play a key role in driving plant growth and energy production [53]. Therefore, further research should clarify whether the change in carbohydrate or phosphorylated sugar levels in rice was related to the changes in the observed plant growth phenotypes.

Interestingly, we found that the basal levels of trehalose were higher in the oePGI lines than in the WT plants, whereas the BPH-induced levels of trehalose (2 days after BPH infestation) were lower in the oePGI plants than in the WT plants (Figure 6D). PGI catalyzes the conversion of F6P to G6P, which can be further converted into glucose-1-phosphate (G1P), and then into UDP-glucose [21]. Both G6P and UDP-glucose are precursors for synthesizing trehalose [54]. Therefore, the enhanced OsPGI1c activity in oePGI lines may increase the G6P and UDP-glucose contents, leading to higher trehalose levels in the oePGI lines compared to the WT plants. It is reported that trehalose plays a significant role in regulating plant defense against piercing–sucking insect herbivores. For instance, trehalose modulates the defense of *Arabidopsis thaliana* and tomato against the green peach aphid (*Myzus persicae*, GPA) by regulating the expression of the key defense gene *PHYTOALEXIN DEFICIENT4* (*PAD4*) and reallocating carbon into starch at the expense of sucrose [55,56]. However, the role of trehalose and its underlying mechanism in regulating rice resistance to BPH require further investigation. On the other hand, the enhanced resistance in the oePGI plants may be due to the allocation of growth-related compounds, such as trehalose, toward the synthesis of defensive compounds in a growth–defense trade-off manner. This could lead to lower trehalose levels in oePGI lines 2 days after BPH infestation.

Carbohydrates, such as sucrose and glucose, have been reported to act as important signaling molecules that influence the levels of phytohormones [57]. For example, Arabidopsis seedlings grown in glucose- or sucrose-containing media exhibit increases in root growth as well as in the levels of JA and ABA [58]. Moreover, the exogenous application of soluble sugars can significantly stimulate ABA accumulation in the embryo axis of yellow lupine (*Lupinus luteus* L. cv. Juno) at all time points after inoculation with *Fusarium oxysporum* f. sp. *lupini* [59]. In tomatoes (*Lycopersicon esculentum* Mill.), glucose application repressed ethylene production by inhibiting ACC oxidase activity [60]. Thus, the observed results—namely, that overexpressing *OsPGI1c* increased the BPH-induced levels of JA, JA-Ile, and ABA but decreased the levels of ethylene—may be due to the change in the levels of soluble sugars in plants. Additionally, H_2_O_2_ has been reported to be negatively regulated by the JA-signaling pathway and ABA in rice [61,62]. Therefore, the decreased levels of H_2_O_2_ may result from the increased JA and ABA levels through their interaction with these pathways. Further research should test this hypothesis.

It has been well documented that ethylene and *OsWRKY45*-dependent SA-signaling pathways negatively regulate the resistance of rice to BPH female adults and/or nymphs [37,63], whereas the JA- and H_2_O_2_-signaling pathways positively modulate the resistance of rice to BPH nymphs [34,36]. Additionally, the ABA-signaling pathway has also been reported to enhance both the resistance of rice to BPH and the hatching rate of BPH eggs [35,64]. Exogenous ABA promotes callose deposition in the rice phloem, which reduces the food intake of BPH and the fecundity of BPH female adults [65]. Moreover, ethylene and ABA-signaling pathways may interact with JA signaling in mediating rice resistance to BPH; for example, Ma et al. [66] reported that the ethylene-signaling pathway regulates rice resistance to BPH attack through the crosstalk with the JA pathway, as a transcriptome analysis revealed that JA and ABA function together in the BPH-induced expression of defense-related transcription factors, contributing to the mechanistic basis of rice resistance to BPH [67]. These crosstalks subsequently lead to the downstream JA-mediated production of defensive compounds such as phenolamides, flavonoids, and volatile terpenes [30,32,36]. Thus, the enhanced resistance of oePGI plants to BPH, the diminished hatching rate of BPH eggs, and the oviposition preference of gravid BPH females are probably associated with the higher levels of JA, JA-Ile, and ABA, as well as lower levels of ethylene, in the oePGI plants compared to the WT plants. Unfortunately, little is known about the mechanism by which *OsPGI1c* activated the JA and ABA pathways and repressed the ethylene pathway during rice defensive processes. Further investigation is required to identify the direct targets of OsPGI1c through protein–protein interaction experiments.

In summary, our results demonstrate that *OsPGI1c* plays an important role in maintaining plant growth and defense, functioning as a moonlighting protein [68]. Upon infestation by BPH, rice plants perceive BPH-derived signals and then activate defense-related pathways, such as those that signal JA and ABA. These changes lead to the up-regulation of defense-related genes and the accumulation of defense compounds, changes that in turn boost the resistance of rice to BPH. The up-regulation of *OsPGI1c* in rice at late stages of BPH infestation both alters the levels of soluble sugars in rice (which not only promotes plant growth but also enhances plant resistance to BPH by strengthening both the JA- and ABA-signaling pathways) and decreases the ethylene-signaling pathway. Our study provides an interesting example of how a single plant gene can synergistically enhance both the tolerance (promoting plant growth) and resistance of plants to herbivores. This study also suggests that *OsPGIc* could serve as a promising molecular target for developing rice cultivars with improved growth and resilience against pests like the BPH through advanced breeding methods, such as gene editing and marker-assisted selection. Additionally, exploring the application of *OsPGIc* in other cereal crops could be a promising approach to broaden its implications for genetic crop breeding.

## 4. Materials and Methods

### 4.1. Plant Materials

The rice (*Oryza sativa*) variety Xiushui110 (XS110, also designated as wild-type (WT)), and two *OsPGI1c*-overexpressing lines (oePGI lines), oePGI-1 and oePGI-2 (see below), were used in this study. The rice seeds of the WT and oePGI plants were pre-germinated in water for two days and then maintained in a growth chamber at 28 °C with a 14 h light and 8 h dark photoperiod. Ten-day-old rice seedlings were transplanted into 30 L blue boxes (length, 50 cm; width, 35 cm; height, 17 cm) and supplemented with a nutrition solution [69]. Thirty-day-old plants were then individually transplanted into 500 mL hydroponic plastic pots (diameter 8.4 cm, height 11.4 cm) supplemented with nutrition solution [69] at 28 °C with a 14 h light and 8 h dark photoperiod. The rice plants were used for further experiments 4-5 days after transplanting.

### 4.2. Insects

The laboratory colony of BPH was originally collected from a rice field located in Hangzhou, China, and has been reared over 10 years on a constant supply of young seedlings of the BPH-susceptible rice variety Taichung Native (TN1). The colony was maintained in a growth chamber at 26 ± 2 °C, with a 12 h/12 h light/dark photoperiod and 80% relative humidity. The TN1 seedlings were replaced every 10 days. Newly emerged BPH adults were collected and transferred to fresh TN1 seedlings at a female–male ratio of 2:1, and gravid BPH females were collected for experiments four days later.

### 4.3. Cloning and Phylogenetic Analysis of OsPGI1c

The full coding sequence of *OsPGI1c* (TIGR ID: Os03g56460) was PCR-amplified from an XS110 cDNA library with specific primers, *OsPGI1c*-F1 and *OsPGI1c*-R1 (Table S2), based on the gene sequences in the Rice Genome Annotation Project database (http://rice.plantbiology.msu.edu/ accessed on 18 December 2024). The PCR products were then cloned into the pMD^TM^ 19-T Vector (TaKaRa, Dalian, China) and confirmed by sequencing. The theoretical molecular weight and predicted isoelectric point of the OsPGI1c protein were calculated using the DNAMAN 8 software (Lynnon Biosoft, San Ramon, CA, USA), and the conserved domain of the OsPGI1c protein was confirmed by InterPro (https://www.ebi.ac.uk/interpro/). The deduced amino acid sequence of *OsPGI1c* was used as a query to identify its closest homologs through the BLASTP program from the National Center of Biotechnology Information (NCBI, http://www.ncbi.nlm.nih.gov). Protein sequences from different plant species sharing high similarity to OsPGI1c were downloaded from NCBI and subjected to multiple sequence alignments using the DNAMAN software (Lynnon Biosoft, San Ramon, CA, USA) with default parameters. For the phylogenetic tree analysis, plant PGI protein sequences were aligned using the ClustalW program in the MEGA X software with the default parameters. The resulting alignment was then used for constructing a neighbor-joining tree using the MEGA X software with 1000 bootstrap replicates [70].

### 4.4. Generation of Transgenic Plants

The full length of the *OsPGI1c* open reading frame was amplified by PCR using the primer pair (*OsPGI1c*-F2 and *OsPGI1c*-R2, Table S2) and fused into the pCAMBIA1301 binary vector to generate overexpression construct pCAMBIA1301-*OsPGI1c* (Figure S4). The resulting construct of pCAMBIA1301-*OsPGI1c* was introduced into the rice variety XS110 through *Agrobacterium tumefaciens*-mediated transformation [71]. Transforming rice, screening homozygous transgenic lines, and identifying the number of insertions were performed as described by Zhou et al. [61]. Two independent homozygous lines, each with a single insertion, designated oePGI-1 and oePGI-2, were selected for subsequent investigation.

### 4.5. Rice Treatments

For the BPH treatment, the rice plants were individually infested by 15 gravid BPH females that were confined within a glass cylinder (4 × 8 cm, with 48 small holes, 0.8 mm in diameter) (Figure S5). Rice plants covered with an empty glass cylinder were used as controls (these were not infested). For mechanical wounding, the lower part of the leaf sheaths (approximately 4 cm in length) was individually punctured 200 times using insect pins. Non-punctured plants were used as controls. For the JA and SA treatments, the 30-day-old seedlings were individually sprayed with 2 mL of 100 µg mL^−1^ JA or 70 µg mL^−1^ SA dissolved in 50 mM sodium phosphate buffer. Each control plant (BUF) was sprayed with an equal volume of 50 mM sodium phosphate buffer. All the samples were immediately frozen in liquid nitrogen and stored at -80 °C until use. There were six independent biological replicates for each treatment at each time interval.

### 4.6. RNA Extraction and qRT-PCR Analysis

To investigate the tissue expression patterns of *OsPGI1c*, various rice tissues, including leaves, inner leaf sheaths, outer leaf sheaths, and roots of 30-old-day seedlings as well as rice seeds, were sampled. For the gene expression analysis of *OsPGI1c* in response to different treatments, rice leaf sheaths from individual plants were collected at 0, 0.5, 1.5, 3, 6, 12, 24, and 48 h post-treatment with six independent biological replicates for each time point. Total RNA was isolated from 100 mg samples using a TaKaRa MiniBEST Plant RNA Extraction Kit and subjected to reverse transcription using a PrimeScript™ RT Master Mix Kit (TaKaRa, Dalian, China) according to the manufacturer’s protocols. The qRT-PCR was performed using the CFX96TM Real-Time System (Bio-Rad, Hercules, CA, USA), with the *ACTIN* gene (TIGR ID: Os03g50885) as the reference that normalizes the expression levels of the target genes. A standard curve was built using a 5-fold diluted cDNA series as templates to calculate the relative expression levels of the target genes. The primers and probes used for qRT-PCR are listed in Table S3.

### 4.7. Phenotypic Analysis

Rice phenotypic parameters of the oePGI and WT lines, including plant height, root length, and the mass of aboveground parts and roots, were measured 30 days after germination in the greenhouse. Plant height was determined from the base of the stem to the top of the flag leaf, whereas root length was measured from the end of the longest root to the base of the stem. The plants were excised at the base of stems, and the aboveground parts and roots were separately weighed. Twenty plants from each line were randomly selected for measurement.

### 4.8. Carbohydrate Measurements

Plants of the oePGI and WT lines were individually infested by 15 gravid BPH females, after which leaf sheaths from individual plants were collected at 0, 2, and 4 days after the BPH infestation and immediately frozen in liquid nitrogen. The carbohydrates were extracted following the method described by Lu et al. [72]. Briefly, 100 mg of finely ground samples in liquid nitrogen was first extracted with 500 µL of 80% (*v*/*v*) ethanol and incubated at 78 °C for 15 min. The pellets were then re-extracted twice with 500 µL of 50% (*v*/*v*) ethanol as above. The supernatants from the three extractions were combined, and carbohydrates in the samples were analyzed using the Waters Xevo TQ-XS triple-quadrupole mass spectrometer (Waters, Milford, MA, USA). A total of 5 μL of each sample was injected into the Waters ACQUITY UPLC BEH HILIC column (1.7 μm, 2.1 × 100 mm). Gradient elution was performed using a mobile phase consisting of solvent A (10 mM ammonium acetate, pH 9.0) and solvent B (90% acetonitrile) at a flow rate of 0.2 mL/min. Negative electrospray ionization mode was used with the following mass spectrometer parameters: capillary voltage, −3 kV; desolvation temperature, 500 °C; desolvation gas flow rate, 1000 L/h; cone gas flow 150 L h^−1^; source temperature, 150 °C. The quantification of each carbohydrate was performed using a linear external standard curve for sucrose, glucose, fructose, and trehalose, respectively. Data were collected using MassLynx V4.2 (Waters, Milford, MA, USA). Each treatment at each time point was replicated 6 times.

### 4.9. Measurement of JA, JA-Ile, SA, ABA, and Hydrogen Peroxide

Plants of the oePGI and WT lines were individually infested by 15 gravid BPH females, after which (at 0, 3, 12, 24, and 48 h post-BPH infestation) the outer leaf sheaths of each plant were harvested and immediately frozen in liquid nitrogen. Frozen samples were ground in liquid nitrogen, and approximately 0.15 g of tissues for each sample was used for phytohormone or hydrogen peroxide analysis.

For phytohormone measurement, endogenous JA, JA-Ile, SA, and ABA were extracted by ethyl acetate spiked with corresponding labeled internal standards (D_6_-JA, D_6_-JA-Ile, D_4_-SA, and D_6_-ABA) and analyzed using an HPLC/mass spectrometry/mass spectrometry (HPLC-MS-MS) system as described in Chen et al. [73]. Six replicates were performed for each treatment at each time point.

To measure hydrogen peroxide, the ground samples were mixed with 1 mL of deionized water and thoroughly vortexed, then centrifuged at 12,000× *g* for 10 min at 4 °C. The supernatant of each sample was used for hydrogen peroxide analysis using the Amplex^®^ Red Hydrogen Peroxide/Peroxidase Assay Kit (Invitrogen, Eugene, OR, USA) according to the manufacturer’s procedures. Six replicates were performed for each treatment at each time point.

### 4.10. Ethylene Analysis

Plants of the oePGI and WT lines were individually covered with a well-sealed glass cylinder (diameter × height, 4 × 50 cm). Then, 15 gravid BPH females were released into the cylinder. Ethylene production was measured by withdrawing 5 mL of gas from the airspace of each cylinder at 12, 24, 48, and 72 h after the start of treatment. Ethylene contents were detected using gas chromatography, as previously described in Lu et al. [74]. Each line was replicated 9 times.

### 4.11. BPH Bioassays

To assess the impact of transgenic plants on the hatching rate and developmental duration of BPH eggs, plants of the oePGI and WT lines were individually infested with 15 gravid BPH females that were allowed to oviposit for 12 h. The number of newly hatched nymphs on each plant was checked and recorded daily until no new nymphs were observed for three consecutive days. All the unhatched eggs on each plant were counted under a microscope to determine the hatching rate. The experiment was repeated 10 times.

To evaluate the effect of overexpressing *OsPGI1c* on the host preference of gravid BPH females, each pair, consisting of a WT plant and an oePGI plant, was covered with a glass cylinder (diameter 4 cm, height 8 cm, with 48 small holes). Then, 15 gravid BPH females were released into the cylinder. After 72 h, the BPH females were removed and the eggs laid on each plant were examined by dissecting all the rice tissues under a microscope. The experiment was repeated 10 times.

### 4.12. Statistical Analysis

All data were statistically analyzed using the IBM SPSS Statistics 26 software (IBM Corp., Armonk, NY, USA). The Student’s *t*-test was used to test the differences in the transcript levels of *OsPGI1c* in response to different stress treatments using rice; the *chi*-square test was used to analyze the differences in host preference of gravid BPH females. The remaining data were analyzed by the one-way analysis of variance (ANOVA), and mean differences were compared using Tukey’s HSD post hoc test (*p* < 0.05). Methods of logarithmic or square-root transformation were applied when necessary to meet the assumptions of normality for ANOVA.

## Data Availability

The data of this study are available from the corresponding author, [Y.L.], upon request.

## Supplementary Materials

The following supporting information can be downloaded at https://www.mdpi.com/article/10.3390/ijms26010169/s1.
