# Supplementary material for: A Cytosolic Phosphoglucose Isomerase, OsPGI1c, Enhances Plant Growth and Herbivore Resistance in Rice
Source: Int J Mol Sci. 2024 Dec 28;26(1):169. doi: 10.3390/ijms26010169 (PMC11720589; doi:10.3390/ijms26010169)
Supplement: Supplementary file 1 [file ijms-26-00169-s001.zip › ijms-3361386-supplementary.pdf]

Supplementary Materials:

A Cytosolic Phosphoglucose Isomerase, OsPGI1c, Enhances Plant Growth and Herbivore Resistance in Rice

Lin Chen, Peng Kuai, Jing Lv, Leilei Li, Yonggen Lou

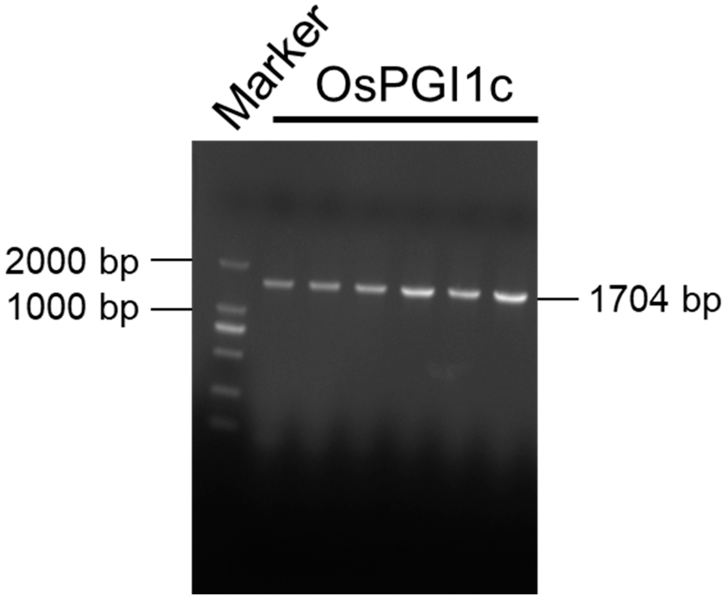

Figure S1. Electrophoresis of PCR-amplified fragments of *OsPGI1c* form a XS110 cDNA library. The PCR-amplified fragments have a length of 1704 bp.

```
1     ATGGCGTCGTCGGCGCTCATCTGCGACACCGAGCAGTGGAAGGGCCTCCAGGCGCATGTTGGGGAGATTCAGAAAACGCACCTGCGCCAT
1      M  A  S  S  A  L  I  C  D  T  E  Q  W  K  G  L  Q  A  H  V  G  E  I  Q  K  T  H  L  R  H
91    CTGATGCATGATGTTGAGCGCTGCAAGGCAATGACAGCTGAGTATGAAGGCATATATCTGGATTACTCGAGGCAGCGTGCGACTGGCGAA
31     L  M  H  D  V  E  R  C  K  A  M  T  A  E  Y  E  G  I  Y  L  D  Y  S  R  Q  R  A  T  G  E
181   ACCATGGAGAAGCTGTTTAAATTGGCCGAGGCTGCAAAGCTCAAGGAGAAGATTGAGAAGATGTTTAGAGGTGACAAGATAAATAGCACA
61     T  M  E  K  L  F  K  L  A  E  A  A  K  L  K  E  K  I  E  K  M  F  R  G  D  K  I  N  S  T
271   GAGAACAGATCAGTGCTTCATGTAGCTCTAAGGGCTCCAAGAGACGAAGTAATAAATAGCAATGGGGTCAATGTGGTTCCCGAAGTTTGG
91     E  N  R  S  V  L  H  V  A  L  R  A  P  R  D  E  V  I  N  S  N  G  V  N  V  V  P  E  V  W
361   GGTGTAAAAGATAAAATCAAGCAATTTTCAGAAACTTTTAGGAGTGGATCATGGGTTGGGGCAACTGGTAAAGCATTGACAAATGTTGTG
121    G  V  K  D  K  I  K  Q  F  S  E  T  F  R  S  G  S  W  V  G  A  T  G  K  A  L  T  N  V  V
451   TCAGTAGGAATAGGTGGTAGCTTTCTTGGTCCTCTGTTTGTGCATGCTGCCCTCCAGACAGATCCAGAAGCTGCAGAATCTGCCAAAGGG
151    S  V  G  I  G  G  S  F  L  G  P  L  F  V  H  A  A  L  Q  T  D  P  E  A  A  E  S  A  K  G
541   CGGCAATTAAGATTTCTTGCAAATGTCGACCCTGTTGATGTTGCACGAAGCATCAAAGATTTAGATCCTGAAACAACACTTGTTGTGGTA
181    R  Q  L  R  F  L  A  N  V  D  P  V  D  V  A  R  S  I  K  D  L  D  P  E  T  T  L  V  V  V
631   GTCTCGAAGACCTTCACAACAGCTGAAACAATGTTAAATGCTCGAACTCTTAAGGAGTGGATTGTCTCTTCTCTTGGACCTGATGCTGTT
211    V  S  K  T  F  T  T  A  E  T  M  L  N  A  R  T  L  K  E  W  I  V  S  S  L  G  P  D  A  V
721   GCAAAACATATGATTGCTGTCAGTACCAATCTTGAGCTTGTGGAGAAGTTTGGAATTGACCCGAAAAATGCTTTTGCATTTTGGGACTGG
241    A  K  H  M  I  A  V  S  T  N  L  E  L  V  E  K  F  G  I  D  P  K  N  A  F  A  F  W  D  W
811   GTTGGTGGCCGCTATAGTGTTTGCAGTGCTGTTGGTGTCCTGCCCTTATCTCTTCAGTATGGCTTTCCGATTGTTCAGAAATTTTTGGAG
271    V  G  G  R  Y  S  V  C  S  A  V  G  V  L  P  L  S  L  Q  Y  G  F  P  I  V  Q  K  F  L  E
901   GGTGCAGCCAGCATCGACAAACACTTTCGTTCATCTTCATTTGAGAAAAATATTCCTGTACTCCTTGGTTTGCTGAGTGTGTGGAATGTT
301    G  A  A  S  I  D  K  H  F  R  S  S  S  F  E  K  N  I  P  V  L  L  G  L  L  S  V  W  N  V
991   TCATTTCTCGGATATCCAGCTAGAGCAATACTGCCCTATTCCCAAGCACTTGAGAAATTTGCACCGCATATTCAGCAGCTTAGCATGGAG
331    S  F  L  G  Y  P  A  R  A  I  L  P  Y  S  Q  A  L  E  K  F  A  P  H  I  Q  Q  L  S  M  E
1081  AGTAATGGAAAGGGTGTCTCCATTGATGGTGTTCAACTGCCCTTTGAGAGTGGTGAAATTGATTTTGGTGAACCTGGAACCAATGGGCAA
361    S  N  G  K  G  V  S  I  D  G  V  Q  L  P  F  E  S  G  E  I  D  F  G  E  P  G  T  N  G  Q
1171  CACAGCTTCTATCAATTAATCCATCAGGGAAGAGTTATTCCTTGTGATTTTATCGGTGTCGTAAAAAGCCAGCAACCTGTTTACTTGAAA
391    H  S  F  Y  Q  L  I  H  Q  G  R  V  I  P  C  D  F  I  G  V  V  K  S  Q  Q  P  V  Y  L  K
1261  GGGGAAATTGTGAGCAATCATGACGAATTGATGTCCAATTTCTTTGCTCAGCCTGATGCGCTTGCTTATGGAAAGACTCCTGAACAACTG
421    G  E  I  V  S  N  H  D  E  L  M  S  N  F  F  A  Q  P  D  A  L  A  Y  G  K  T  P  E  Q  L
1351  CATAGCGAGAAAGTACCTGAACATCTTATCCCTCATAAGACTTTTCAGGGCAACCGACCATCGCTTAGTTTATTGCTGCCCTCATTATCT
451    H  S  E  K  V  P  E  H  L  I  P  H  K  T  F  Q  G  N  R  P  S  L  S  L  L  L  P  S  L  S
1441  GCTTATGAGATTGGACAGCTTTTAGCCATCTACGAGCACCGGATTGCAGTCCAGGGTTTCCTATGGGGAATAAACTCATTTGACCAGTGG
481    A  Y  E  I  G  Q  L  L  A  I  Y  E  H  R  I  A  V  Q  G  F  L  W  G  I  N  S  F  D  Q  W
1531  GGAGTGGAACTGGGCAAGTCTCTTGCCTCTCAAGTGAGAAAATCTCTACATGCATCCCGCGTTGAAGGAAAGCCTGTCCTGGGGTTTAAC
511    G  V  E  L  G  K  S  L  A  S  Q  V  R  K  S  L  H  A  S  R  V  E  G  K  P  V  L  G  F  N
1621  AGCAGTACTACAAGTTTGCTGACACGATATCTTGCTGTTGAGCCATCCACTCCTTACAACACTACCACACTGCCGAAAGTTTGA
541    S  S  T  T  S  L  L  T  R  Y  L  A  V  E  P  S  T  P  Y  N  T  T  T  L  P  K  V  *
```

Figure S2. Full-length CDS and deduced amino acid sequence of *OsPGI1c*. * represents the stop codon. The underlined red and blue sequences denote the two sugar isomerase (SIS) domains.

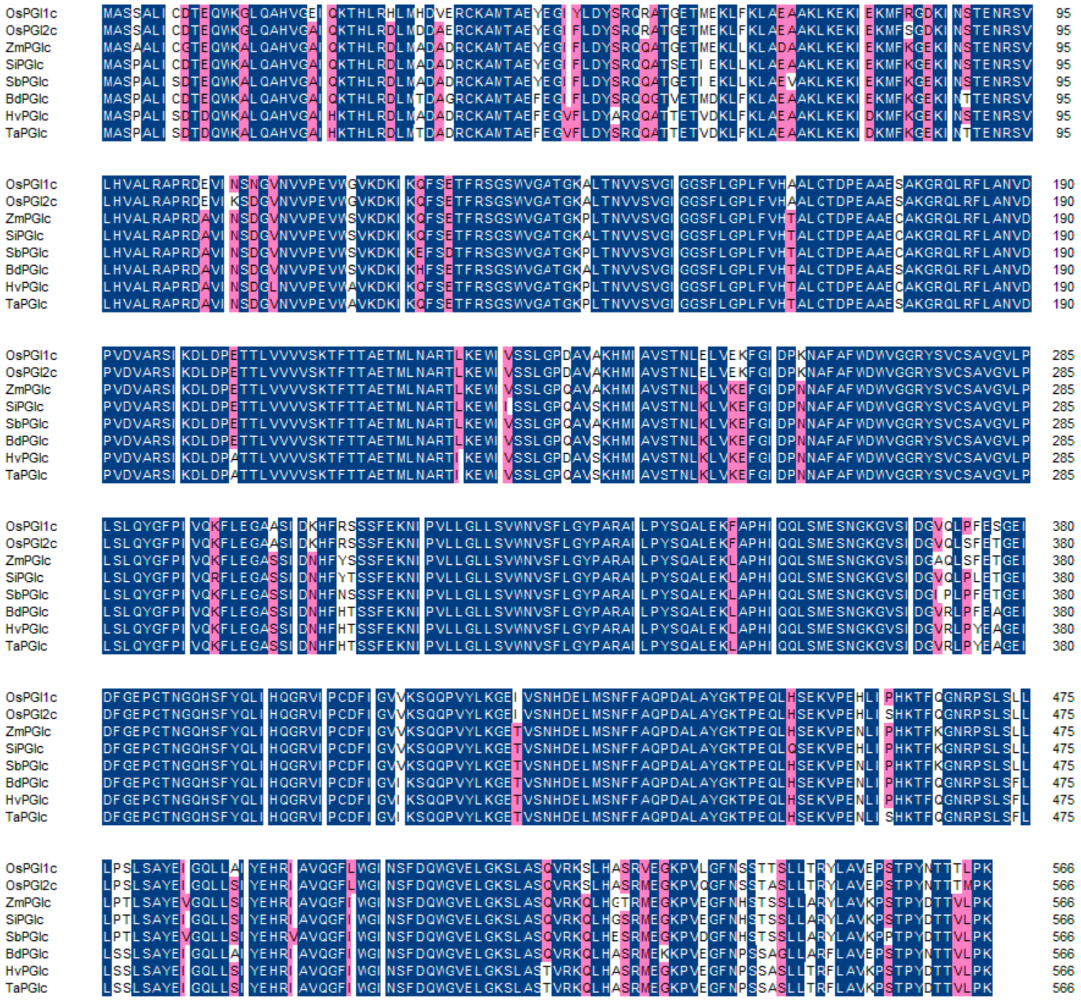

Figure S3. Amino acid sequence alignment of OsPGI1c and its closely clustered homologues. Fully and partially conserved amino acids are shaded by blue and pink, respectively.

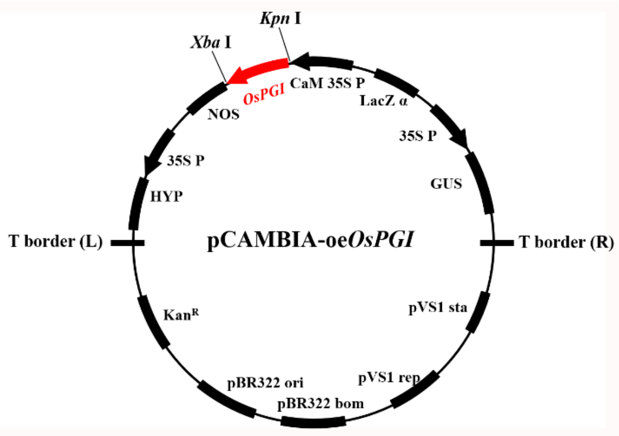

Figure S4. Construction of the plant overexpression vector pCAMBIA-oe*OsPGI*. The expression of *OsPGI* was driven by the CaMV-35S promoter.

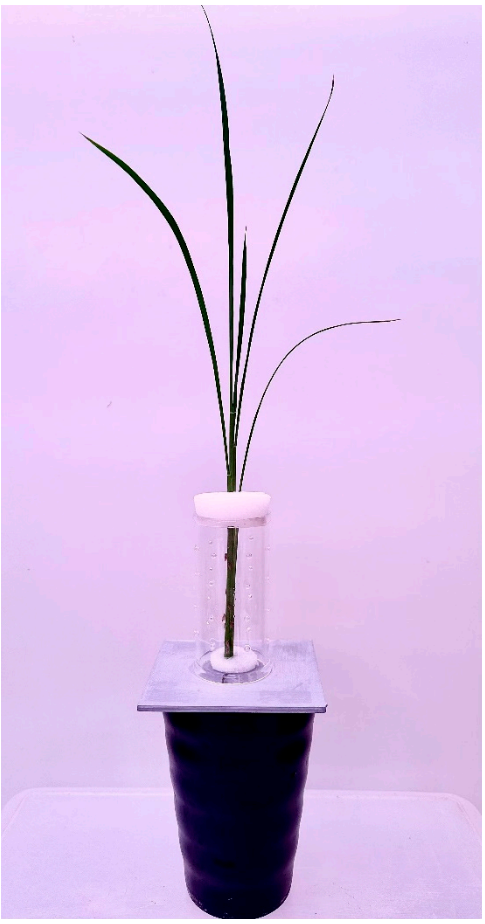

Figure S5. The experimental setup involved rice plants infested by 15 gravid BPH females that were confined within a glass cylinder.

Table S1. Detailed information of plant PGI proteins used for phylogenetic analysis.

Protein name	Plant species	Predicted subcellular localization	Accession number
OsPGI1c	*Oryza sativa*	cytosol	XP_015632584.1
OsPGI2c	*Oryza sativa*	cytosol	XP_015644261.1
ZmPGIc	*Zea mays*	cytosol	NP_001105368
SiPGIc	*Setaria italica*	cytosol	XP_004981572
SbPGIc	*Sorghum bicolor*	cytosol	XP_021304536
BdPGIc	*Brachypodium distachyon*	cytosol	XP_003569006
HvPGIc	*Hordeum vulgare*	cytosol	ABE41789
TaPGIc	*Triticum aestivum*	cytosol	ABE41790
CxPGI1c	*Clarkia xantiana*	cytosol	P54240
CwPGIc	*Clarkia williamsonii*	cytosol	P54239
CmPGIc	*Clarkia mildrediae*	cytosol	P54237
CcPGI1c	*Clarkia concinna*	cytosol	P54235
CxPGI2c	*Clarkia xantiana*	cytosol	P54242
CcPGI2c	*Clarkia concinna*	cytosol	P54241
RcPGIc	*Ricinus communis*	cytosol	XP_002510371
SlPGIc	*Solanum lycopersicum*	cytosol	NP_001265881
HaPGIc	*Helianthus annuus*	cytosol	XP_021983676
SoPGIc	*Spinacia oleracea*	cytosol	XP_021862407
GmPGIc	*Glycine max*	cytosol	NP_001242028
AtPGIc	*Arabidopsis thaliana*	cytosol	NP_001332180.1
AhPGIc	*Arabidopsis halleri*	cytosol	BAC77717
OsPGIp	*Oryza sativa*	plastid	XP_015611795
AtPGI1	*Arabidopsis thaliana*	plastid	AK227111
RcPGIp	*Ricinus communis*	plastid	XP_002514018
SoPGIp	*Spinacia oleracea*	plastid	XP_021861839

Table S2. Primers used for cloning of *OsPGI1c*

NO.	Name	Sequence (5′-3′)
1	OsPGI-F1	ATGGCGTCGTCGGCGCTC
2	OsPGI-R1	TCAAACTTTCGGCAGTGTGG
3	OsPGI-F2	GGGGTACCATGGCGTCGTCGGCGCTC
4	OsPGI-R2	GCTCTAGATCAAACTTTCGGCAGTGTGG

Table S3. Primers and probes used for qRT-PCR of target genes

Gene	TIGR ID	Primers (5′-3′)	Probe (5′-3′)
ACTIN	Os03g50885	FP: TGGACAGGTTATCACCATTGGT	HEX-CGTTTCCGCTGCCCTGAGGTCC
		RP: CCGCAGCTTCCATTCCTATG	
OsPGI	Os03g56460	FP: AAATTTGCACCGCATATTCA	FAM-CCATCAATGGAGACACCCTTTCCA
		RP: ACCACTCTCAAAGGGCAGTT	
